# Bilayered graphene/*h*-BN with folded holes as new nanoelectronic materials: modeling of structures and electronic properties

**DOI:** 10.1038/srep38029

**Published:** 2016-11-29

**Authors:** Leonid A. Chernozatonskii, Viсtor A. Demin, Stefano Bellucci

**Affiliations:** 1 Emanuel Institute of Biochemical Physics of RAS, 119334 Moscow, Russian Federation; 2 INFN– Laboratori Nazionali di Frascati, Via E. Fermi 40, 00044 Frascati, Italy

## Abstract

The latest achievements in 2-dimensional (2D) material research have shown the perspective use of sandwich structures in nanodevices. We demonstrate the following generation of bilayer materials for electronics and optoelectronics. The atomic structures, the stability and electronic properties of Moiré graphene (G)/h-BN bilayers with folded nanoholes have been investigated theoretically by ab-initio DFT method. These perforated bilayers with folded hole edges may present electronic properties different from the properties of both graphene and monolayer nanomesh structures. The closing of the edges is realized by C-B(N) bonds that form after folding the borders of the holes. Stable ≪round≫ and ≪triangle≫ holes organization are studied and compared with similar hole forms in single layer graphene. The electronic band structures of the considered G/BN nanomeshes reveal semiconducting or metallic characteristics depending on the sizes and edge terminations of the created holes. This investigation of the new types of G/BN nanostructures with folded edges might provide a directional guide for the future of this emerging area.

Two-dimensional structures are attractive materials for application use, because they have many unique properties. For example, graphene – a plane of hexagonally arranged carbons - has such properties as high strength and thermal conductivity, as well as a high mobility of charge carriers. However, it has a significant disadvantage, i.e. the lack of energy gap, which makes its use in electronics extremely limited. The band structure varies depending on the number of layers, their mutual orientation^1^ and the presence of periodically arranged holes^2^,^3^,^4^.

It is well known that when bilayer graphene or graphite is cut by electron beam, their edges are connected^5^,^6^. This is the result of the chemical activity of edge C-atoms. If these holes are periodically arranged in the hexagonal lattice, the bilayer mesh has energy gap up to 0.43 eV for the smallest holes^4^. It is possible to obtain nanotubes by the electron beam, because of the fact that bilayer massive areas can be connected^7^. Holes with sizes 1.4–7.4 nm in bilayered graphene were obtained by K. He *et al*.^8^ from a monolayer, which is typically covered with a surface layer of hydrocarbons. Heating to 800 °С removes surface adsorbates to leave clean graphene. The electron beam was focused to create nanopores, then surface adsorbates are converted into a stable secondary layer after cooling to room temperature. Multiple papers (see for a review ref. 9) are devoted to the preparation of large-area graphene nanomeshes (GNM) by now. In particular, I. Jung *et al*.^10^ have successfully fabricated high-density GNM using a platinum nano-network as a pattern mask. A simpler method has been presented by top-down lithographic techniques by using block copolymer nanospheres^11^ or interferometer method^12^.

Hexagonal boron nitride (*h*-BN) is another monolayer material which is a dielectric analogue of graphene. It has some advantageous properties: higher thermal and chemical stabilities than graphene, mechanical robustness, resistance to oxidation and good optical properties^13^. Note that *h*-BN nanomeshes were grown on crystal surfaces using different techniques^14^,^15^, however it was not yet possible to receive samples of freestanding monolayer meshes. Recently, Y. Liao^16^ has presented a scalable method for the fabrication of holes in a BN monolayer using silver catalytic nanoparticles.

Hybrid analogues of graphene and *h*-BN have also attracted intensive interest, because of their commensurate structural parameters and distinct electronic properties. It is possible to form BN-C covalent connective parts in nanotubes^17^, in-plane^18^ or sandwich^19^,^20^ heterostructures. The similar synthetic route for producing G/BN NMs provides a low-cost and simple way to fabricate G/BN structures with folded nanoholes, hence studying these structures is necessary for their future applications.

Therefore we assume that it will be possible to fabricate G/BN meshes with folded holes by the aforementioned methods. Nowadays, precisely aligned graphene are grown on *h*-BN in a controllable manner^21^.

Both graphene and *h*-BN are single-layer materials and have the same hexagonal lattice. Differences are in their composition and inter-atomic distance (1.42 Å in graphene and 1.45 Å in boron nitride^22^). When layers are put onto each other, a disparity leads to the formation of structures such as Moiré patterns in bilayered graphene. One of the layers can rotate by the twist angle θ relative to the other. The hexagonal unit supercell of the Moiré pattern with θ = 0° consists of 10182 atoms and their parameter is λ = 72.50 Å. The supercell can be decreased by rotation of one layer relative to the other one. The Moiré parameter is determined by the relation^20^: 
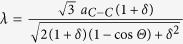
, 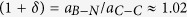, 0° ≤ θ ≤ 30°). The following two cases are typically considered. The first case is when the center of rotation is selected in the area where the atom in a single layer is right above the atom in the other layer. The second case is when the rotation center is situated in a middle of the C-atomic hexagon in the carbon layer, coinciding with the center of the atomic BN hexagon of the other layer. Formula λ = (θ, δ) practically does not change in both of these cases.

Moiré graphene/h-BN structure consists of some different areas^23^. Two of them look like AA- and AB- stacking areas, similar to Moiré graphene bilayer. The existence of interfaces connecting graphene and h-BN boundaries has been proved by Raman, AFM and TEM characterization^18^.

In these bilayer connected structures of all sp^2^-hybridizated atoms, the number of non-hexagonal rings in each inner torus region of a hole must satisfy a relation extracted from Euler theorem formula, when pentagons are absent: n_7_ + 2n_8_ = 12, where n_7_ and n_8_ are the numbers of heptagons and octagons^24^. The inner part of a high symmetry AA hole consists of six octagons whereas the inner part of a lower symmetry AB hole includes heptagons.

The main types of G/BN two-layer materials with periodically arranged folded holes presented in this work have been modeled by the DFT method. Only symmetrical forms of holes formed in the AA (or AB) centers are considered here. Naturally, the asymmetric shape of the holes should result in a severe disruption of symmetry and corresponding changes in the electronic properties of graphene as in the case of single-layered nanomeshes^25^. This work provides a directional guide for engineering of graphene/BN nanomeshes and investigating their fundamental properties, as well as device applications.

## Results and Discussions

### Moiré 6.4° patterns

**Figure 1:**
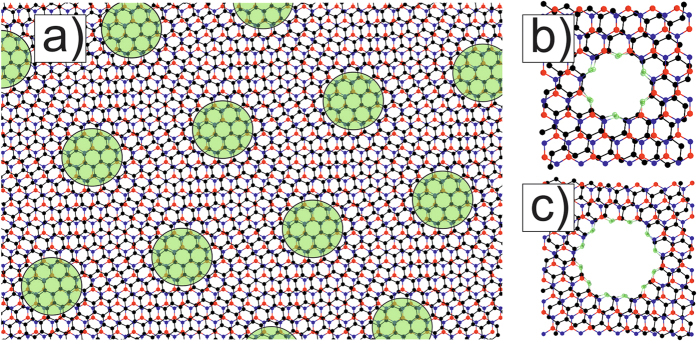
Moiré G/BN structure with θ = 6.4° (**a**), and scheme of creation of two ≪round≫ holes in AA-stacking green areas. Green atoms on the edges of different layers are connected by covalent bonds: 6 pairs for a R_1_ hole (**b**) and 12 pairs for a R_2_ hole (**c**). The boron, nitrogen and carbon atoms are presented by red, blue and black balls, respectively.

We consider Moiré structure θ = 6.4°, whose unit cell (BN)_67_C_146_ consists of 280 atoms (Fig. 1) and has Moiré parameter λ = 20.7 Å. AA-stacking area is a part of the Moiré structure where each atom in one layer is located practically over the atom of another layer. AB-stacking Bernal areas are situated between each three AA-areas (Fig. 1a). Holes can be produced after irradiation by local e-beam similarly to Ref. 8. Here we have considered the holes only in AA- and AB- stacking areas where we can create simple ≪round≫ forms. These energetically favorable holes have covalently connected zig-zag edges (green atoms in Fig. 1b,c) similar to the observed zig-zag bonded edges of bilayered graphene^5^,^6^. The six AB-areas are around each AA-area (Fig. 1): three with C-N pair in the area center, and the next alternate three areas with C-B pair in their centers.

### Holes in AA-stacking areas

A ≪round≫ hole is formed by recessing the hexagonal pieces from the AA-area center of the corresponding Moiré structure (Fig. 1b,c). The inner part of a hole is a torus with topological octagon defects of atoms. Holes with radius 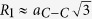 are formed by extraction of *N*_*q*_ = 6(2*q* − 1) + *N*_*q*−1_ atoms from each layer (q = 1, 2…). The G/BN Moiré meshes with such ≪round≫ holes will be denoted as Mθ{AA_o_q}.

**Figure 2:**
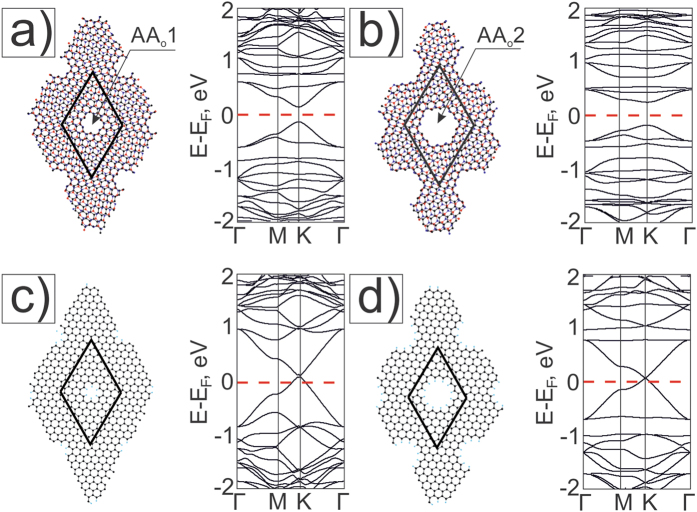
Atomic models (left views) and their electronic band structures (right views) of nanomeshes: (**a**) M6{AA_o_1} and (**b**) M6{AA_o_2}, (**c**) and (**d**) their separate graphene components GNM_o_1 and GNM_o_2, respectively.

At first, we study the M6{AA_o_1} structure with the smallest holes (Fig. 2a), when one hexagon is removed from every layer in the centers of AA regions. The supercell (BN)_64_C_140_ consists of 268 atoms and has superlattice parameter *λ* = 20.66 Å. Covalent bonding of the layers leads to the changes in the band structure of corresponding G and BN nanomeshes. C-N and C-B bond have different length that causes breaking the graphene sublattice symmetry. This leads to opening of a direct band gap E_g_ = 0.28 eV. Larger holes (Fig. 2b) are potential barriers and they impede the movement of electrons, therefore holes augmentation leads to increase of the value of the band gap to E_g_ = 0.43 eV. Bonds between atoms are stressed in holes, so the lattice constant decreases to λ = 20.58 Å. We remark that it is simpler to form bigger AA-holes with radius 
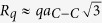
 (q-integer >1) in Moiré structures with smaller angles θ, because the distance between joined atoms enlarges with the increasing of angle θ (see Supplementary Materials I).

Electronic band structures of the meshes M6{AA_o_} with holes of radius R_1_ and radius R_2_ are shown on the right in Fig. 2a and b.

It is interesting to compare these electronic spectra with the band structures of corresponding monolayer graphene nanomeshes (GNM_o_1, GNM_o_2) with the same ≪round≫ hole structures and the same supercells (Fig. 2c,d). These GNMs with hydrogen on the edges have indexes (8, −9; 9, −1) in terms of Dvorak *et al*.^26^, where the GNM energy gaps are defined by the parameters of unit supercells. Indexes (n_1_, m_1_; n_2_, m_2_) correspond to the indexes of two unit supercell vectors in the unit cell vectors of graphene. According to the rules in the paper [26] our GNMs are metallic with the view very similar to graphene band structure. However the Fermi level is a little lower than in graphene, because of electron localization near the edges. Hydrogen atoms on the GNM hole edges do not break the symmetry of graphene nanomesh sublattices.

**Figure 3:**
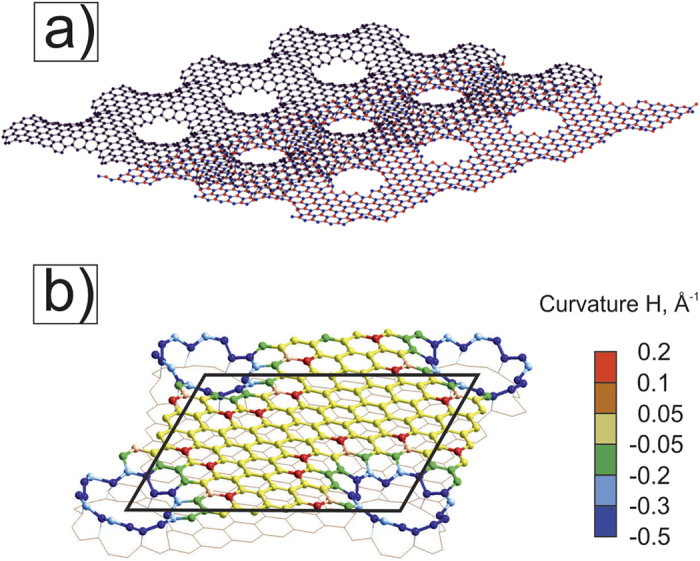
General view of the structure M6{AA_o_2} and separate layers. BN part (bottom layer) remains flatter than graphene (top layer) - (**a**). Geometrical measures for mesh M6{AA_o_1} - (**b**), the unit cell is shown as parallelogram.

Graphene is more flexible compared to the BN layer. Therefore the graphene surface after the G/BN nanomesh formation is more convex in the parts between the holes than the same BN surface area. This is clearly seen in the general view of the mesh M6{AA_o_2} (Fig. 3a).

For the meshes M6{AA_o_1} and M6{AA_o_2} the variations in Z coordinates of C atoms and B (N)-atoms are equal to δ_C,B(N)_ ≈ 1.96, 1.18 (1.21) Å and δ_C,B(N)_ ≈ 2.32, 0.99 (1.03) Å, respectively, in the regions outside of the inner torus parts (the Z-axis is perpendicular to the layers). Thus, the structure corrugation of the graphene part of the considered G/BN meshes can explain the Dirac point splitting in contrast to the electron bond spectra of monolayer GNMs (Fig. 2). We calculated the local curvature around each atom of the graphene part according to formula 7 from A.A. Pacheco Sanjuan’s paper^27^. Positive curvature of the graphene changes from 0.5 to 0.2 Å^−1^ (Fig. 3b). Central large area has a curvature close to zero. We see some correlation between the curvature picture and electronic band structure of the bilayered meshes. So the M6{AA_o_1} gap has smaller value than the M6{AA_o_2} structure where graphene local curvature has smaller central H ≈ 0 area. Therefore, the smaller distance between the holes, the larger graphene part bends and it has larger positive and negative H local areas. This can lead to increase of the gap value E_g_.

### Holes in AB-stacking areas

**Figure 4:**
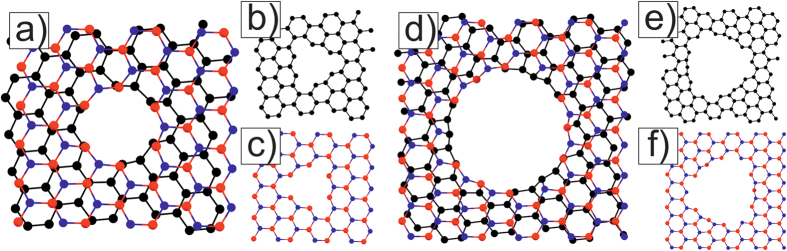
Scheme of atoms connection in the AB-area with a C-B pair in the center of the G/BN Moiré M6. The smallest AB-hole (**a**) is obtained from two triangle holes in graphene (**b**) and *h-*BN (**c**) layers. A bigger hole (**d**)–stems from two truncated triangle holes in each layer (**e,f**). Red–boron, blue–nitrogen, and black–carbon atoms.

Here, we would like to describe the creation of ≪round≫ holes in G/BN bilayer in AB-stacking areas. The smallest ≪round≫ hole with radius 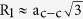 can be obtained by excluding the pair of atoms from the AB area center and the closest three pairs. Then, each separate layer will have a triangle-like hole (Fig. 4). If we extract four pairs from the AB area center with C-B atoms, we will obtain a ≪round≫ hole with radius 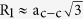 (Fig. 4a). Here, six C-atoms on the border of the triangle graphene hole (Fig. 4b) connect with six B-atoms on the border triangle BN hole (Fig. 4c). By extracting next closest six C-B pairs and next twelve C-N pairs, we will obtain a round hole with 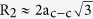. Here twelve edge C-atoms (Fig. 4e) connect to nine B-atoms and three N-atoms on the border of the truncated triangle hole in the BN-layer (Fig. 4f). Notably, similar triangular holes in the BN-layer with a predominance of the boron atoms at the boundary were observed earlier in the experiments^27^,^28^.

The bigger holes with radius 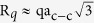 (q-integer > 1) and number of extracted atoms *N*_*ext*_ = 2*N*_*q*_, *N*_*q*_ = 6(2*q* − 1) + *N*_*q*−1_ in AB area can be created in Moiré structures with smaller angles 0° ≤θ < 6°.

**Figure 5:**
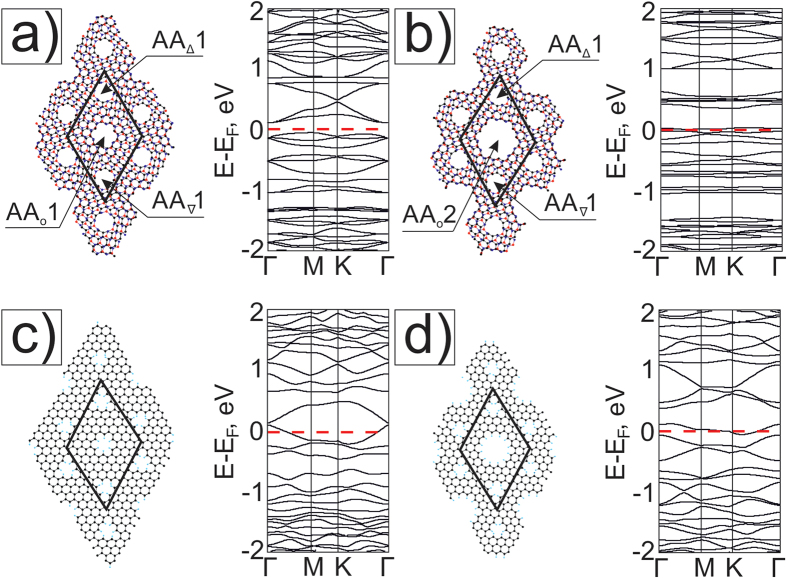
Atomic models and their electronic band structures of nanomeshes based on G/BN Moiré 6.4° (**a**) - M6{AA _o_1, AB_∇Δ_1} with holes in AA areas and ∇ and Δ holes in AB areas, (**b**) - M6{AA _o_2, AB_∇Δ_1} with increasing holes in AA areas and the same ≪round≫ ∇ and Δ holes in AB areas, and of the corresponding monolayer graphene nanomeshes GNM_o_1,_∇Δ_1 and GNM_o_2,_∇Δ_1 - (**c**) and (**d**).

We also consider three holes in the supercell of the mesh by the adding of two holes in AB-stacking areas of the nanomesh M6 with AA-≪round≫ holes (Fig. 5). After forming such holes, the band structure is drastically changed with respect to the case of AA-≪round≫ hole meshes (Fig. 2). Almost straight branches are formed near E_F_ that shows strong localization of electrons on all holes (Fig. 5a,b). The formation of additional holes in AB-areas of the supercell even changes the graphene superlattice symmetry. Strong electron localization around AB_∇_1 and AB_Δ_1 holes with radius R_1_ (Fig. 5a) and the large curvature of graphene in the region between the holes (fragments of half-nanotubes of small diameter) lead to flattening of the branches. The spectrum of the mesh M6{AA_o_1, AB_∇Δ_1} contains a Dirac point rising above E_F_ and the energy gap E_g_ = 0.16 eV (Fig. 5a). A miniband of the mesh M6{AA_o_2, AB_∇Δ_1} is formed above the actual gap (Fig. 5b) which is higher and bigger than in the case of the mesh M6{AA_o_1, AB_∇Δ_1} (Fig. 5a). Corresponding graphene nanomesh band structures lose the aspect of the graphene electronic structure too (Fig. 5c,d). The shift of the E_F_ leads to the appearance of the hole conductivity in GNM_o_1,_∇Δ_1 and GNM_o_2,_∇Δ_1 structures. The graphene nanomeshes with chosen supercell (8, −9; 9, −1) retain the metallic properties.

It is remarkable that if one compares Fig. 5b and d, the strained structure M6{AA_o_2, AB_∇Δ_1} has a 0.32 eV second band gap in the Γ point above the conductivity zone, whereas we obtain the smaller value of gap (0.25 eV) over E_F_ in the corresponding GNM_o_2,_∇Δ_1.

### θ = 10.9° Moiré patterns

Further, we continue to study the formation of closed holes in the Moiré pattern θ = 10.9° whose supercell (BN)_27_C_55_ consists of 110 atoms. In such a small unit cell, only one ≪symmetric≫ hole from three possible ones can be formed in one of the AA, AB_B_ or AB_N_ centers. Two types of AB areas are situated between AA-regions: AB_B_ and AB_N_ with B- atom and N- atom in their centers as it has been shown earlier.

**Figure 6:**
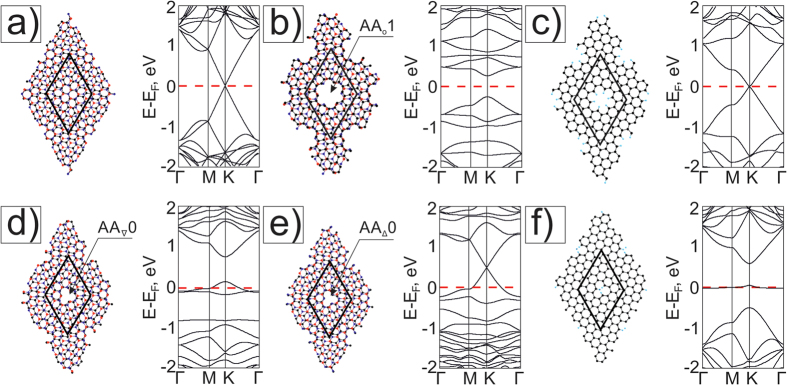
Atomic (left views) and electronic band structures (right views): (**a**) Moiré pattern θ = 10.9°, and (**b**) M11{AA_o_1} with ≪round≫ holes in AA areas (the hole is formed by the hexagon extraction of six atoms for each layer G/BN supercell); (**c**) the corresponding GNM_o_1; (**d**) M11{AA_∇_0} with triangle _∇_0 holes in AA areas (one nitrogen and one carbon extracted atoms), (**e**) M11{AA_Δ_0} with triangle _Δ_0 holes in AA areas (one boron and one carbon extracted atoms), (**f**) GNM_∇_0 (1 carbon atom is extracted from the G supercell). The role of graphene deformation in band structure changing of G/BN mesh was estimated in Supplementary Materials III on the example of the disconnected from M11{AA_o_1} graphene part.

Firstly we consider atomic and electronic structures of Moiré meshes M11 with holes in AA-areas (Fig. 6). As it is shown on Fig. 6a, the unstrained G/BN Moiré 10.9° has a graphene-like band structure in the (−1 eV, 1 eV) energy region with the Dirac point. When we form a ≪round≫ hole in the supercell by extracting C- and BN hexagons from the AA-area, we get the strained structure M11{AA_o_1} similar to the strained mesh M6{AA_o_1} and opening a gap in its band structure (Fig. 6b). Charge distribution shows the non-equivalence of edges carbon atoms (see Supplementary Materials II). M11{AA_o_1} has a smaller flattened area of the graphene layer and a higher curvature near the holes than M6{AA_o_} structures. It is one of the factors influencing the change of the band gap in the spectra of the considered G/BN nanomeshes. The separated graphene part GNM with the supercell (6, −2; 2, 4) in terms of [26] has metallic property (Fig. 6c).

The cases when two atoms are removed from the AA-area center are considered below. We can construct materials with different electronic characteristics if B-C or N-C atom pairs are deleted. It is possible to create in the AA-area ≪triangle≫ holes of the _∇_q type. We can make the smallest _∇_0 holes after extraction of two atoms only (Fig. 6). The extracted carbon atom in the G layer or B (or N) atom from the BN layer is a regular vacancy defect. The presence of B (or N) atoms on the hole edges (Fig. 6d) leads to the formation of a mini-zone with localized branches near E_F_ (Fig. 6d,e). The corresponding graphene nanomesh with (6, −2, 2, 4) unit cell has a narrow metallic minizone near E_F_ (Fig. 6f).

In this work only the simplest meshes Mθ{AA(AB)_∇_0} with point-like _∇_0 holes have been studied. We will considered later for a more detailed description of meshes with _∇_q (q > 0) holes.

**Figure 7:**
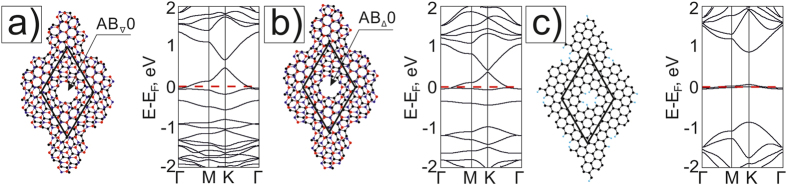
Atomic schemes (left views) and electronic band structures (right views) of meshes: M11{AB_∇_1} with 4С, B and 3N extracted atoms in the center of the AB-area (**a**) and M11{AB_Δ_1} with 4C, N and 3B - extracted atoms in the other AB-area (**b**), corresponding GNM_∇_1 with (6; −2; 2; 4) unit cell (**c**).

Finally, we have modeled a new type of ≪round≫ holes in the AB-regions of BN/G bilayers which can be organized also by knocking the atoms with an electron focusing beam with nm point on the AB- area of a bilayer (see for example [8]). A ≪round≫ hole can be formed by knocking 8 atoms (Fig. 7). The closed hole edges are formed using the connection of zig-zag edges of unbonded layers of BN/G mesh. One hole is formed when B + 3 N and 4C atoms are extracted from layers (Fig. 7a) or when N + 3B and 4C atoms are extracted (Fig. 3b). As a result, six atom pairs are connected and form all sp^2^ smooth folded hole with radius 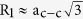. The holes in the top BN layer have a triangular shape (the same triangular holes were considered earlier in GNMs^28^,^29^). The hole in the bottom G layer has the same shape but it is rotated by 180° similarly to Fig. 4. Each hole forms a torus-like structure containing topological defects of six atomic octagons.

The electronic band structure of mesh M11{AB_∇_1} with six B atoms on each hole edges has metallic character. Electron doping of the BN part opens the gap on the Dirac point of carbon branches situated at 0.3 eV up Fermi level – Fig. 7a. There Metallic behavior also occurs in the case of mesh M11{AB_Δ_1} (Fig. 7b). Transformation of the electron density happens near the holes, and the energy gap opens in the band structure above E_F_ in the K-point. The corresponding graphene nanomesh GNM_∇_1 of the (6; −2; 2; 4) unit cell with triangle holes is metallic with a narrow metallic band near the Fermi level (Fig. 7c), which is in agreement with GNMs with triangle holes^28^.

Remark that we chose symmetrical holes in G/BN NMs based on many experiments that proved evidence of mainly symmetrical holes in graphene, as well as in h-BN by catalytic preparations^18^ and electron beam irradiation^30^,^31^. A. W. Robertson *et al*.^30^ observed a hexagonal R_1_ hole when six atoms are extracted from graphene, and H.J. Park^31^ also formed hexagonal nanoholes in mono- and bilayer h-BN. We hope that in the nearest future the facility of automatic nano manipulation for cutting 2D materials by electron beam will enable one to realize G/BN structures with folded nanoholes, also including the creation of the proposed periodic structures.

### Main parameters of G/BN nanomeshes

Table 1 lists the main parameters of Moiré meshes M6 and M11. It is worth to note that the total energy of nanomeshes s found to have values close to G/BN Moiré structures. For example: the creation of the same AA_o_1 hole in M11 and M6 leads to energy gains of 13 and 75 eV/cell relatively to the unconnected case. Their unit cell parameters L are somewhat smaller than the corresponding parameters λ of G/BN Moiré unit cells. All considered bilayer structures with folded holes are energetically stable.

## Conclusion

In this paper we predict new 2D-material nanomeshes based on Moiré graphene/hexagonal boron nitride bilayers. We have demonstrated the creation models of different types of folded holes in AA- and AB-stacking areas of the Moiré bilayers. These perforated bilayers may present electronic properties different from the properties of monolayer structures, such as graphene, h-BN and their nanomeshes^26^,^32^. Contrary to graphene nanomeshes, BN/G meshes have holes with edges which are non-chemically active. Here, carbon atoms are covalently bonded to the edges of the BN mesh component. Therefore, they are more inert to the environment. The distortion and symmetry breaking of the graphene part lead to semiconductive properties of G/BN meshes with AA-≪round≫ holes. The value of the E_g_ is defined by the shape and size of the periodically arranged holes. Some of the calculated G/BN meshes with holes in AB-areas are metallic. The energy band gap E_g_ in the bilayer NM G/BN increases due to symmetry breaking in the curvature graphene component and due to the charge transport at the toroidal border of the hole compared with a similar graphene mesh. Our estimations show that energy gap values should be higher than experimentally observed energy band gap of the GNM, i.e. E_g_ = 30 meV at room temperature^12^. This advantage is important primarily for semiconducting devices. Thus G/BN structures with folded nanoholes can be suggested as novel 2D materials for application in electronic and photonic. Unlike single graphene structures, BN monolayer is highly piezoelectric^33^ because it is noncentrosymmetric. Such engineered G/BN NMs can be a candidate material for various nanoelectromechanical applications. In particularly G/BN structures with folded nanoholes can be used as a nanosensor of molecules. As a result of compression, which happens with application of an electric field, round holes turn into ovals. This action can lead to delays of molecules (such as DNA) into the hole. The current flow of the graphene component of G/BN mesh can control those portions of the molecule that get stuck in the hole.

Thermally induced rotation of graphene on hexagonal boron nitride has been found recently^34^. This confirms the possibility of the formation of G-BN nanomeshes on the base of Moiré structures with a prescribed configuration.

We also believe that the establishment of the considered holes with closed edges can be formed in G/BN structures with triangle-like holes (see Figs 3, 4, 5, 6 and 7). It is possible due to the fact that such triangular holes with a predominance of the boron atoms at the boundary layer, were already observed in 2007^35^, specifically in the BN layer. Similar form holes were observed in graphene^36^,^37^. It should be also interesting to investigate the magnetic properties of these nanomeshes with a prevalence of one of the atoms (B or N) on the edges of the hole. Theory shows their important role in the existence of the magnetic moment in monolayered meshes^35^, because they display substantial magnetism in proportion to the number of unpaired electrons of N (or B) atoms along the zig-zag edges. It should be also interesting to observe how the considered G/BN nanomesh properties are changed under the influence of external magnetic fields. It is known^38^ that the compression of graphene bilayers also leads to a change of the electronic properties. There will be compression or extension of a graphene/BN nanomesh under external electric field, since the BN layer has piezoelectric properties of the graphene part, because it determines electronic properties of the whole bilayer mesh in general. We will investigate these properties in subsequent works.

Entire devices could be engineered in G/BN bilayer materials through the appropriate combination of different folded hole types. Such hybrid structures will pave the way for the development of future two-dimensional electronic and electro-optical devices.

### Method of calculation

First-principle calculations within the framework of density functional theory were performed using SIESTA Package^39^. Ab initio calculations with standard norm-conserving pseudo-potentials, flexible numerical LCAO double zeta + polarization orbital basis sets were used. The 2D system is separated from its periodic images by a vacuum distance no less than 20 Å. The exchange-correlation potential was incorporated by using the local density approximation (LDA) due to Perdew–Zunger^40^ for electronic structure calculations. To calculate the equilibrium atomic structures, the Brillouin zone was sampled according to the Monkhorst–Pack scheme^41^ with a k-point’s density 0.06 Å^−1^. Structural relaxation was carried out until the change in the total energy was less than 10^−4^ eV, or forces acting on each atom were less than 10^−3^ eV Å^−1^. Note that this method has a drawback-underestimation of the band gap of about 25–40%^42^. We understand that magnetic order can have influence on the electronic properties of monolayer graphene or BN nanomeshes, but tests show that the influence is not strong and the difference in total energy of non-, ferro and antiferromagnetic states is less than 0.02 eV/cell (see Supplementary Materials IV).

## Additional Information

**How to cite this article**: Chernozatonskii, L. A. *et al*. Bilayered graphene/*h*-BN with folded holes as new nanoelectronic materials: modeling of structures and electronic properties. *Sci. Rep.* **6**, 38029; doi: 10.1038/srep38029 (2016).

**Publisher's note:** Springer Nature remains neutral with regard to jurisdictional claims in published maps and institutional affiliations.

## Supplementary Material

Supplementary Information

## Figures and Tables

**Table 1:** Number of atoms in the calculated supercells, their total energies and geometric parameters.

	N_total_ atoms	N(C)	N(B)	N(N)	E_total_, eV	E_g_, eV	L, Å
M11	110	56	27	27	−18328.6	0.0	12.97
M11{AA_o_1}	98	50	24	24	−16301.0	0.30	12.85
M11{AA_∇_0}	108	55	27	26	−17889.2	0.0	12.93
M11{AA_Δ_0}	108	55	26	27	−18075.7	0.0	12.92
M11{AB_∇_1}	102	52	26	24	−16783.8	0.0	12.90
M11{AB_Δ_1}	102	52	24	26	−17156.1	0.0	12.89
M6	280	146	67	67	−46571.3	0.0	20.72
M6{AA_o_1}	268	140	64	64	−44546.2	0.28	20.67
M6{AA_o_2}	232	122	55	55	−38516.9	0.43	20.58
M6{AA1_o_, AB_Δ∇_1}	252	132	60	60	−41833.1	0.16	20.56
M6{AA2_o_AB_Δ∇_1}	216	114	51	51	−35798.8	0.01	20.50
