# Supplementary material for: Bilayered graphene/h-BN with folded holes as new nanoelectronic materials: modeling of structures and electronic properties
Source: Sci Rep. 2016 Nov 29;6:38029. doi: 10.1038/srep38029 (PMC5126688; doi:10.1038/srep38029)
Supplement: Supplementary Information [file srep38029-s1.pdf]

Bilayered graphene/*h*-BN with folded holes as new nanoelectronic materials: modeling of structures and electronic properties:

Supplementary Information

Leonid A. Chernozatonskii[1*], Victor A. Demin[1], Stefano Bellucci[2]

[1] Emanuel Institute of Biochemical Physics of RAS, 119334 Moscow, Russian Federation

[2] INFN– Laboratori Nazionali di Frascati, Via E. Fermi 40, 00044 Frascati, Italy

I. Relationship between Moiré angle θ and connection energy of layers in G/BN nanomeshes with AA holes.

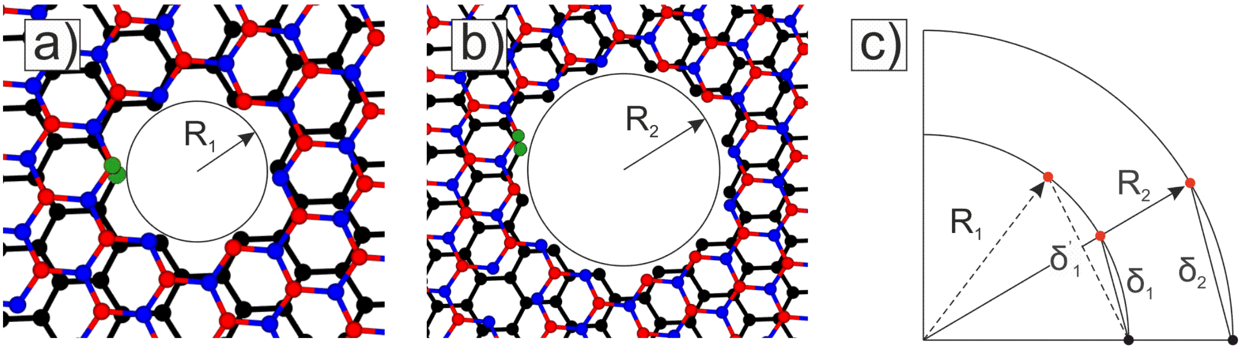

Fig. S1. Unconnected holes AA_o1 (a) and AA_o2 (b) in the Moiré bilayer with angle θ=6.4° and scheme (c) of the atom location after one layer rotating.

Let's consider holes AA_o1 and AA_o2 in the BN-G bilayer with Moiré angle θ=6.4° before covalent layers connection (Fig. S1a,b). Atoms in a hole oscillate while the structure is heated, which leads to the convergence of hole edge atoms of layers and then to connection of the neighbor atom pairs marked green in Fig. S1. When projection δ of the distance between joined atoms (Fig. S1c) gets bigger, the structure with folded holes should be tenser, i.e. it needs

to be treated with higher temperatures in order to connect atoms (similar to graphene edges connection[1]). Therefore connection energy $\Delta = E_{after} - E_{before}$ must depend on the initial distance between atoms in the pairs. Here, E_{before} and E_{after} are total energies of the structures before atom connection and after connection process, respectively. For example, the creation of the same AA_o1 holes in meshes M11 and M6 leads to energy Δ gains: -13 and -75 eV/cell, according to DFT calculations. The corresponding projections have the difference $\delta_{11} - \delta_6 = 0.2$ Å as follows from Table 1. Thus, when projection δ is decreased by reducing angle θ, the connection energy also decreases.

	R_1=2.46 Å	R_2=4.92 Å	R_3=7.38 Å
θ	δ, Å		
6.4	0.27	0.55	0.82
10.9	0.47	0.93	1.40
15	0.64	1.28	1.93

Table S1. The projection of distance between of the joined atoms δ for different Moiré angle θ and hole radius R_n.

II. Charge density calculations

Charge density calculations of the M11{AA_o1} structure were made in the frame of SIESTA program. The results presented on four clipping planes (Fig. S2a) show: 1) decrease of graphene symmetry is due to nonequivalence of C-N and C-B bonds and charge distribution on the inside of the hole - (Fig. S2b; 2) the positive charges location are concentrated on the crossed plane N-atoms of the hole inset – Fig. S2c; 3) negative charge focuses on the crossed plane C-atoms connected with N- atoms (positive charges are focused near the C-atoms connected with B- atoms) - Fig. S2d; 4) the C-atoms on the next ring on the clipping plane of Fig. S2e have negative charges. These pictures clearly demonstrate the charge transfer from BN to graphene parts of the M11{AA_o1} structure.

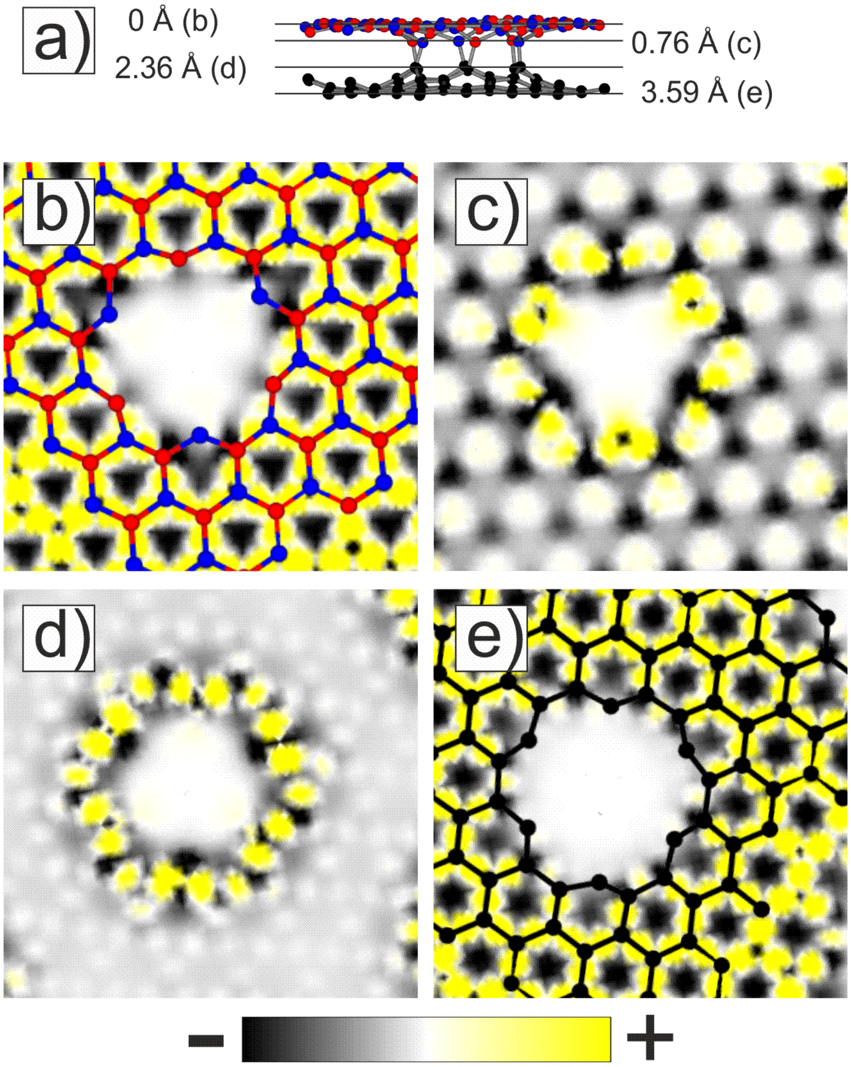

Fig. S2. Side view of mesh M11{AA_o1} positions of clipping planes(a), and charge redistribution on each plane (b-e).

III. Comparison of band structures of the separated G part with its reconstructed analogue and the whole structure of NM11{AA_o1}

Consideration of a graphene layer separated from the BN layer in mesh NM11{AA_o1} has been made to understand the nature of a band gap opening in the bilayer G/BN nanomesh. The band structure of the graphene corrugated part with hydrogen atoms on the hole edges without optimization of the carbon mesh (Fig. S3a) shows a semiconducting behavior with the gap E_g=0.03 eV (Fig. S3b). The difference in the Z-coordinates of carbon atoms in the structure leads to symmetry breaking. This reflects a small band gap opening and distinguishes it from the NM11R{AA_o1} band gap E_g=0.5 eV (Fig. S3c). Therefore, the model shown in Fig 3a does not take into account the contribution of such strain into the big gap opening for the mesh. We must remark that on the other hand, flatted GNM11R1 where all edge atoms are equivalent has the Dirac point in the electron spectrum (Fig. S3d) as this corresponds to the rule[2] for this GNM.

We think that the main reason for the big gap opening in the NM11R{AA_o1} spectrum is redistribution of the electron density in both layers during their deformation. This is indicated by the fact that deformations of the BN layer can play an important role for the band gap value[3].

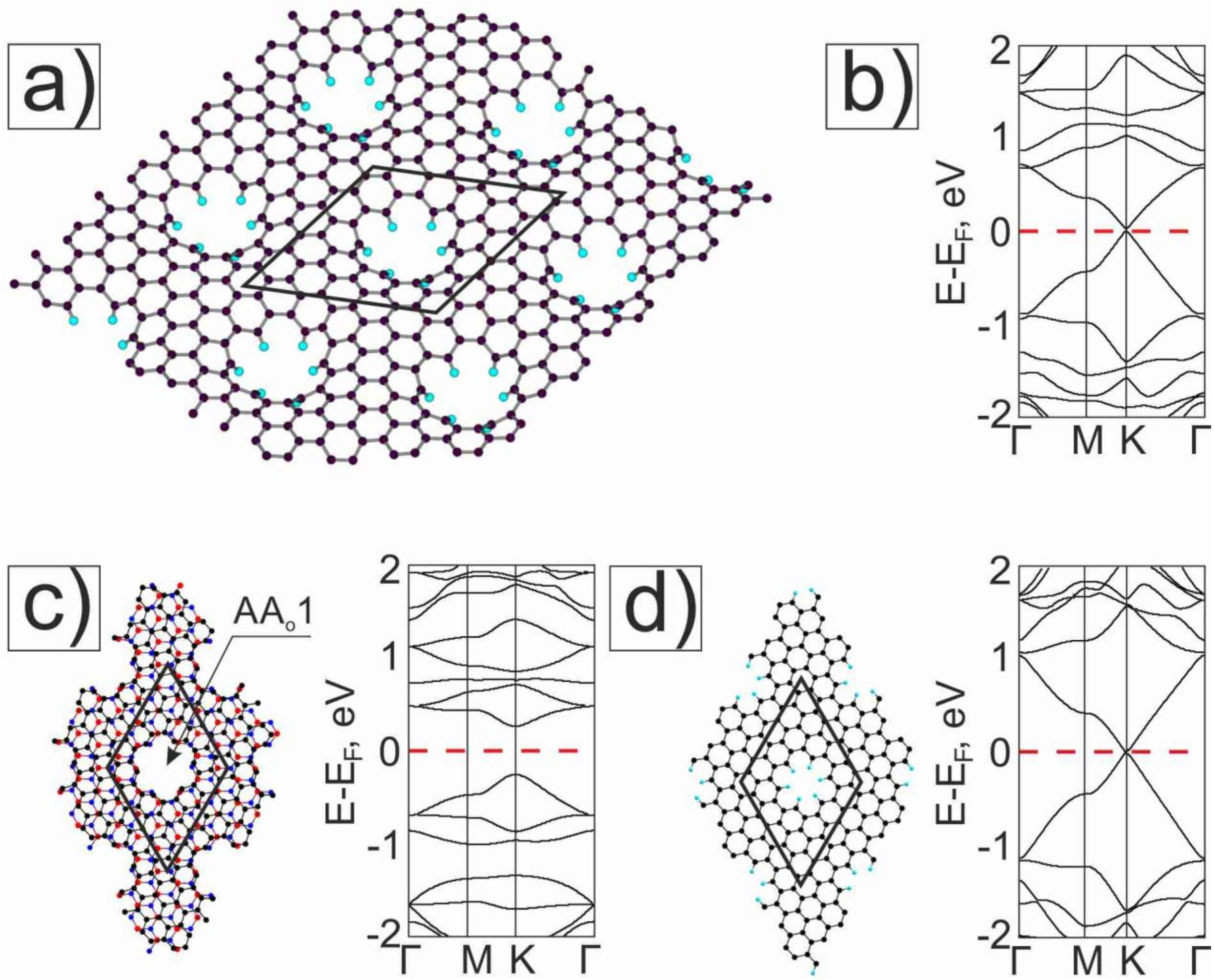

Fig. S3 Comparison of the electronic band structure of the single layer graphene nanomesh (after removing the BN layer and hydrogenating the dangling C atoms from NM11{AA_o1}) (a,b). The bilayer nanomesh M11{AA_o1} (c) and the optimized graphene nanomesh (d).

IV. Spin-polarized calculations

Magnetic order plays an important role in monolayer BN and G nanomeshes with triangular holes[4]. In order to understand the role of spin polarization in the G/BN structures we have made the appropriate calculations for the structures M11{$AA_\nabla0$} and M11{$AA_\Delta0$} with triangle holes. The considered meshes have different numbers of boron and nitrogen atoms. As one can see from Fig. S4, their band structures are not significantly changed for ferro- or antiferromagnetic states of the meshes.

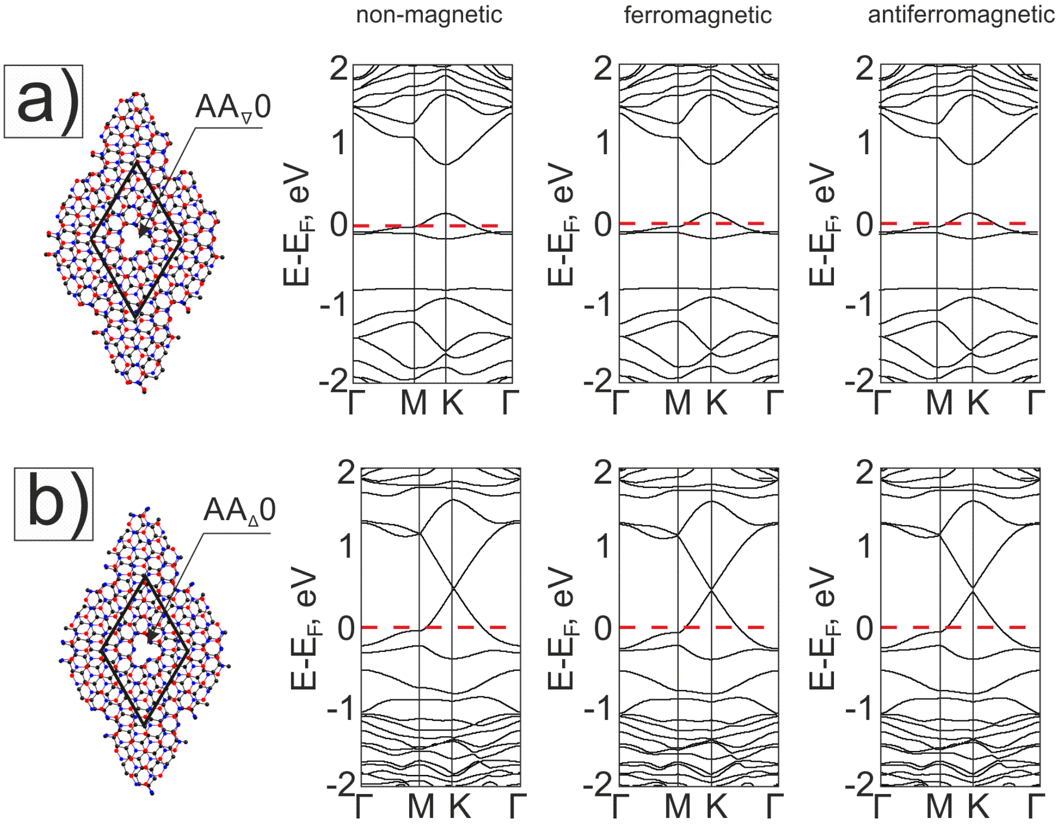

Fig. S4 Comparison of electronic band structures in different magnetic states of M11{$AA_{\nabla}0$} and M11{$AA_{\Delta}0$}.

Energy calculations show that the energy difference is very small also for the magnetic order types (less than 0.02 eV /unit cell - Table 2).

Nanomeshes	Non-magnetic, eV	Ferromagnetic, eV	Antiferromagnetic, eV
M11{$AA_{\nabla}0$}	-17889.17	-17889.15	-17889.15
M11{$AA_{\Delta}0$}	-18075.69	-18075.69	-18075.70

Table 2. Total energies of M11{AA□0} and M11{$AA_{\Delta}0$} unit cells in non-magnetic, ferro- and antiferromagnetic states.

1. Jia, Z. et al. Graphene edges: a review of their fabrication and characterization. *Nanoscale* **3**, 86-95(2014)

2. Dvorak, M., Oswald, W. & Wu, Z. Bandgap Opening by Pattering Graphene. *Sci. Rep.* **3,** 2289 (2013).

3. Wu, J., Wanga, B., Weia, Y., Yangb, R. & Dresselhaus, M. Mechanics and Mechanically Tunable Band Gap in Single-Layer Hexagonal Boron-Nitride. *Mater. Res. Lett.* **1**, 200–206 (2013).

4. Ouyang, F., Yang, Z., Xiao, J., Wu, D. & Xu, H. Electronic Structure and Chemical Modification of Graphene Antidot Lattices. *J. Phys. Chem. C* **114**, 15578-15583 (2010).
